# Machine part data with part-of relations and part dissimilarities for planted partition generation

**DOI:** 10.1016/j.dib.2022.108065

**Published:** 2022-03-21

**Authors:** Daniel Bakkelund

**Affiliations:** Institute of Informatics, University of Oslo, TechnipFMC, PO Box 1080, Oslo 0316, Norway

**Keywords:** Machine parts, Part-of relations, Dissimilarity, Planted partition, Clustering, Link recovery

## Abstract

Identifying relationships between entities in data is a central topic across various industries and businesses, from social networks to supply chain and heavy manufacturing industries. In this paper we present data from a database of machinery represented in terms of machine parts. The machine parts are originally organised in tree structures where the vertices are machine part types, and the edges are “part-of” relations. Hence, each tree represents a type of machinery broken down into its machine part constituent types. The data we present is the union over these trees, making up a directed acyclic graph describing the type hierarchy of the machine parts.

The motivation for publishing the dataset is the following real-world industry problem: Each tree represents a mechanical design, and over time some designs have been copy-pasted with minor modifications. The new instances have been given new identifiers with no reference to where from they were copied. In hindsight, it is desirable to recover the copy-paste links to for interchange between essentially identical designs. However, telling which parts are copies of which other parts has turned out to be difficult. In particular, the metadata has a tendency of displaying higher similarities within a composite part than between a part and its copy. Due to non-disclosure, we cannot provide the metadata, but we provide element wise dissimilarities that are generated based on the metadata using classical methods such as Jaccard similarity on description texts, material types etc. The dissimilarities are obtained from a data science project in the company owning the data, trying to tackle the very problem of recovering the copy-paste links.

Availability of labeled data on this data set is limited, so based on our in-depth knowledge of the problem domain, we present a data synthesisation method that can generate arbitrarily large problem instances of the copy-paste problem based on the sample data, that provides a realistic representation of the real world problem. The problems are presented as planted partitions of vertices of directed acyclic graphs with vertex dissimilarities, and thus constitutes a typical classification problem along the lines of graph- or network clustering.

The type of industry data we present is usually company confidential, bound by intellectual property rights, and generally not available to scientists. We therefore publish this anonymised dataset to offer real world sample data and generated problem instances for researchers that are interested in this type of classification problems, and on which theories and algorithms can be tested.

The data and the problem generation methodology are backed by a Python implementation, providing both data access and an API for parameterised problem generation. The data is also available as raw files.

## Specifications Table

**Table:** 

Subject	Data Science
Specific subject area	Applied Machine Learning
Type of data	Table of machine parts types and part-of relations Table of dissimilarities between machine parts types Python code
How the data were acquired	Extraction from company database.
Data format	Analysed Raw
Description of data collection	**Machine part types:** The machine part data is extracted from a relational database. The initial raw data is organised as trees of machine parts, where each node has a unique identifier and a type-id. In the data collection process, the trees have been replaced by a graph of types as follows. The vertices of the graph corresponds to the set of types of the tree vertices. Then, edges are added to the graph if there is an edge in a tree between vertices of corresponding types. The resulting graph thus represents the *type part-of structure* defined by the trees. Since one type of machinery may be a part of different types of high level machinery, in the way a type of tire may be a part of many types of cars, the type hierarchy becomes a graph, rather than a tree. And moreover, since a part cannot another part of the same type [7], the type hierarchy is a directed acyclic graph.^1^
	Our particular subset of machine part types was chosen as follows. When we generated the above graph for all machine parts, we found that the graph had one very large connected component and a large set of disconnected vertices, but also eight connected component in the range of 11 to 40 vertices. Since each of these connected component closely correspond to single designs, we chose these eight connected components as our sample dataset.
	**Dissimilarities:** The dissimilarity data is obtained from an internal project in the company owning the data trying to tackle the very problem of recovering the mentioned copy-paste links. The dissimilarities are generated based on metadata about the machine part types, such as description texts, material types, weights etc.
Data source location	Proprietary database owned by TechnipFMC,^2^ a privately held company in the Oil & Gas sector.
Data accessibility	Repository name: Mendeley Data
	Data identification number: 10.17632/dhhxzdzm3v.1
	Direct URL to data: https://data.mendeley.com/v1/datasets/dhhxzdzm3v/
Related research article	D. Bakkelund, Order Preserving Agglomerative Hierarchical Clustering, Mach Learn. (2021). doi:10.1007/s10994-021-06125-0.

## Value of the Data

• The type of data we present is usually company confidential, and therefore very difficult to come by for researchers. By publishing a small subset of the data together with code that can proliferate the data based on our understanding of the problem domain, we hope to allow other researchers to test their hypotheses and methods on close to real world data.
  In this respect, we particularly mention the problem of *order preserving clustering* [1]. This is a field in development where there are currently no public datasets available for benchmarking and/or testing of methods and hypotheses. We therefore wish to publish this dataset, and the model for generating classification problems from this dataset, to support further development of this new branch of classification research.
• Since we present dissimilarity data with additional relations, the main audience is likely to be researchers and practitioners within classification and clustering that work with data that has additional structure. As a non-exhaustive list of examples we mention graph- and network clustering [5], order preserving clustering [3], acyclic partitioning [4] and clustering with constraints [2].
• One of the contributions of this paper is a model for generation of planted partitions simulating the copy-paste problem. The model is based on our in-depth knowledge about the problem domain, and we believe that this model, together with the published data, provides realistic representations of the previously described copy-paste problem. Hence, models and algorithms that perform well on these planted partitions can be expected to perform well also on the real dataset.
• As for the mentioned industry problem, this is an *excess inventory problem* in that the machine part manufacturer has an increasing amount of machinery in stock. A traditional approach to excess inventory is that of *excess inventory disposal* [8] to free up capacity. However, this is easily sub-optimal for expensive machinery, both with respect to economy, and also with respect to the environment, as manufacturing of complex steel based machinery has a large ${\text{CO}}_{2}$ footprint. Rather, TechnipFMC states that if they can match similar machinery in the described fashion, then this will lead to increased sales from inventory, rather than producing new machinery. Thus, yielding a double up-side compared to decimating the machinery in stock.

## 1 Data Description

This section describes the format of the flat files containing the machine part data and the dissimilarity data. Note, however, that the data is also available through the provided python API.

The data is available as two CSV^3^ files, one file for the machine part structures, and one for the machine part dissimilarities.

### 1.1 The machine part file

The machine part file is named parts.csv. Each line in the machine part file is formatted as

〈id 〉 (,〈child id 〉)*

That is, each line is a comma separated list of integers. The first integer is the part type identifier (id), and the remaining integers (if any) are the part types that occur as “part-of” type id.

For example, if the data was constituted by the graphwhere the arrows indicate that 1 and 2 are both *part-of* 0 and that 2 is *part-of* 3, then the corresponding file would look like

$$\begin{array}{c} 0,1,2\\ 1\\ 2\\ 3,2 \end{array}$$

### 1.2 The dissimilarities file

The dissimilarities are also organised in a CSV file, dissimilarities.csv, with each line on the format

〈integer:a〉,〈integer:b〉,〈decimal number:d〉

Here, the integers are part type ids, as given in parts.csv, and the dissimilarity d is the dissimilarity between the specified types. The indices are always ordered so that a<b, and for the dissimilarities, we always have 0≤d≤1.

Given the above example part type structure, a corresponding dissimilarity file would be on the form

$$\begin{array}{c} 0,1,0.2021\\ 0,2,0.3141\\ 0,3,0.2718\\ 1,2,0.1414\\ 1,3,0.7071\\ 2,3,0.2600 \end{array}$$

That is, the dissimilarity between 0 and 1 is 0.2021, meaning 0 and 1 are more dissimilar than, say, 1 and 2 that has a dissimilarity of 0.1414.

### 1.3 Some statistics on the connected components

The part data constitutes eight connected components, where each connected component is a small DAG. Table 1 lists some typical graph statistics for the connected components.

**Table 1:** Some key characteristics of the connected components of the machine parts dataset: cc no., the index of the connected component; cc, size the number of vertices in the connected component; The in/out deg, the directed average degree of the connected component; p, the probability that for a pair of random vertices a and b, the edge (a,b) exists in the transitive reduction.

cc no.	cc size	in/out deg.	p
0	12	0.92	0.17
1	14	0.93	0.14
2	13	0.92	0.15
3	40	1.27	0.07
4	20	1.35	0.14
5	11	1.18	0.24
6	20	1.10	0.12
7	20	0.95	0.10

*Note:* The table is an adaptation of Bakkelund [1, Table 2].

### 1.4 Parent-child dissimilarities

**Fig. 1:**
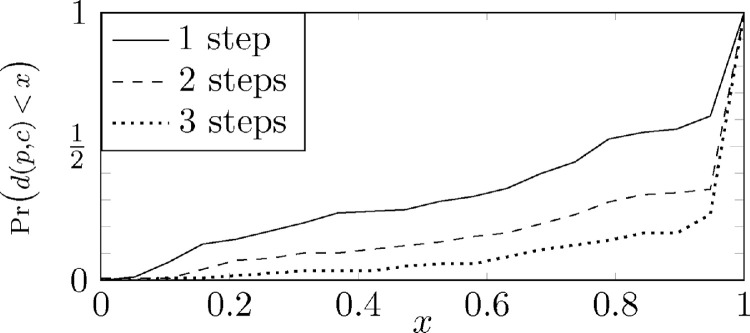
Probability for a part and a sub-part to have dissimilarity no higher than x. The first axis value is the dissimilarity in the range [0,1], and the second axis is the probability of a parent part p and child part c to have a dissimilarity d(p,c) no higher than x; that is, Pr(d(p,c)<x). The curves represent parent-child pairs that are separated by 1, 2 or 3 levels. We see that there is a higher probability for low dissimilarity between a part and a contained part if the containment is direct (1 step) compared to a nested containment (≥2 steps).

A feature of the dissimilarity data, is that there is a high probability for the dissimilarity of a part and a sub-part to be low. This is due to a significant overlap in metadata, stemming from the fact that a part and its sub-parts are often closely related in several ways. For example, machinery wrought out of steel will often have parts with similar material- and mechanical properties. For machinery that will be used under harsh environmental conditions, the environmental characteristics of the parts must necessarily be very similar. Description texts describing a sub-part will often contain references to the containing part, and so on. Deducing the dissimilarities based on this metadata therefore sometimes lead to low dissimilarity between parent and child. For the copy paste problem, this is a complicating factor, since a part and a sub-part can never be copy-paste related. The dissimilarity distributions between parts and sub-parts are displayed in Fig. 1.

## 2 Experimental Design, Materials and Methods

In this section, we describe the model for generation of planted partitions based on the published dataset. The model is a simplified representation of the copy-paste mechanism in the problem domain. An important goal of the model has been to keep it simple, while at the same time not underplaying the complexity of the copy-paste process and the following changes to the data.

It should be noted that our notion of a planted partition is not the same as the probabilistic concept of planted partitions sometimes encountered in clustering literature [6]. Rather, in the model we present, the generation of the planted partitions is based on our understanding of the copy-paste problem, and our wish to simulate this. We still choose to refer to this as *planted partitions*, since they are, in name, exactly that.

On a high level, the model works as follows. Given a connected component C from parts.csv, the dissimilarities from dissimliarities.csv, a positive integer n, a location parameter μ and a scale parameter $\sigma^{2}$, we generate a planted partition with n+1 parallel instances through the following steps:

1. Make n copies of C, providing us with the connected components ${\left\{C_{i}\right\}}_{i=0}^{n}$ where $C=C_{0}$. Denote the vertices of C by $\left\{v_{1}^{0},\ldots ,v_{m}^{0}\right\}$, and similarly denote the vertices of $C_{i}$ by $\left\{v_{1}^{i},\ldots ,v_{m}^{i}\right\}$ so that $v_{k}^{i}$ is the copy in $C_{i}$ of $v_{k}^{0}$.
2. For every connected component $C_{i}$, define the *intra component dissimilarities*as follows:
  $$d\left(v_{r}^{i},v_{s}^{i}\right)=d_{0}\left(v_{r}^{0},v_{s}^{0}\right),$$
  where $d_{0}$ is the dissimilarity found in dissimilarities.csv. That is, the intra component dissimilarities in the copies are the same as in the original connected component.
3. Let $Y∼N(\mu ,\sigma^{2})$ be a random variable where $N(\mu ,\sigma^{2})$ is the Gaussian distribution located at μ with variance $\sigma^{2}$. We define the stochastic function α:[0,1]→[0,1] by α(x)=x+Y through rejection sampling, naively continuing to draw from Y until x+Y∈[0,1]. The *inter component dissimilarities* may now be defined as
  $$d\left(v_{r}^{i},v_{s}^{j}\right)=\alpha (d_{0}\left(v_{r}^{0},v_{s}^{0}\right)).$$
  That is, we distort the dissimilarity between the copy-paste instances by adding Gaussian noise.

The result is a set of machine parts with part-of relations that is the union of all the copies $C_{i}$ equipped with dissimilarities. The corresponding planted partitions are the sets $P_{k}={\left\{x_{k}^{i}\right\}}_{i=0}^{n}$ for 1≤k≤m, defining the m sets of copy-paste elements.

**Fig. 2:**
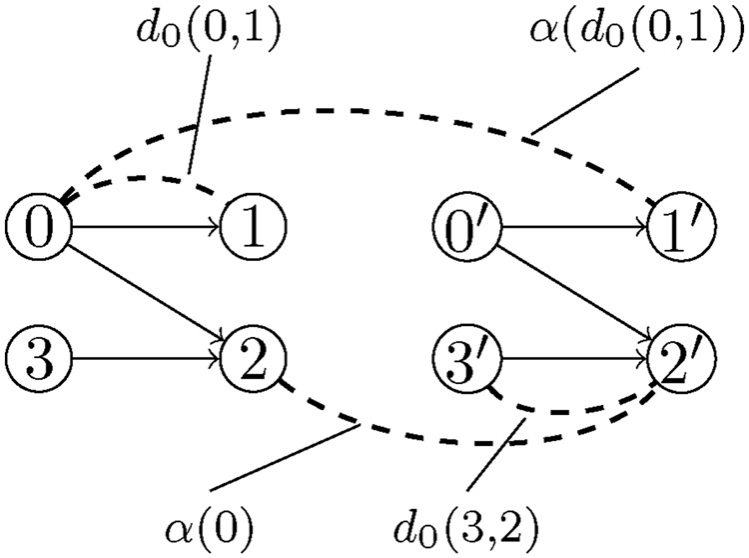
The dashed lines indicate dissimilarity links. We can see that the dissimilarity between 2 and the copy $2^{\prime }$ is α(0), the dissimilarity between the element 0 and the copy of the child $1^{\prime }$ is $\alpha (d_{0}\left(0,1\right))$, which is the perturbed dissimilarity of $d_{0}\left(0,1\right)$. And finally, that the intra-component dissimilarity between $3^{\prime }$ and $2^{\prime }$ is identical to that in the original connected component, namely $d_{0}\left(3,2\right)$.

An example is depicted in Fig. 2.

The model is subject to at least two simplifications that deviate from the real world case. In both cases, we chose to do this to keep the model simple. However, we also believe that this does not compromise the problem generation in terms of benchmarking relative the real world problem:

• *The topology of the original and the copy is identical.*
  We do not add or remove vertices or relations when we copy. In the real application, this happens to some extent.
• *The intra component dissimilarities are unchanged when copied.*
  Since metadata is changed after copying, the intra component dissimilarities will also change in the real application.

We summarise the input and output of the planted partition generation in Tables 2 and 3.

**Table 2:** Table of inputs to the planted partition generation process.

Parameter	Explanation
cc-ids	The ids of the connected components that shall be duplicated
n	The number of copies to make
μ	The mean translation of the dissimilarities under α
$\sigma^{2}$	The variance in the noise applied by α

**Table 3:** Table of outputs from the planted partition generation process.

Data	Explanation
X	A set of vertices X making up the union of the original connected components as well as all the copies
E	A set of edges (a,b)∈X×X denoting all the part-of relations of both the original connected components as well as the copies
d	A dissimilarity measure defined on all of X generated according to the above procedure
$P={\left\{P_{i}\right\}}_{i=1}^{\left|X\right|}$	The planted partitions; that is, the sets consisting of machine parts that are copies of each other.

Now, given a generated problem instance (X,E,d,PP) and a classification procedure C, to which degree can C recover P if given only X, E and d?

## 3 Python Implementation

An open source python implementation of the above model is made available. The library is most easily installed via PyPi by

python3 -m pip install machine-parts-pp [--user]

Notice that the library requires python version 3.0 or higher. For further documentation of the provided functionality, please visit https://pypi.org/project/machine-parts-pp/.

## Ethics Statements

N/A.

## CRediT authorship contribution statement

**Daniel Bakkelund:** Data curation, Methodology, Software, Writing – review & editing.

## Declaration of Competing Interest

The authors declare that they have no known competing financial interests or personal relationships that could have appeared to influence the work reported in this paper.

## Footnotes

1 If a part contains another part of the same type as a sub-part, this leads to a cycle of containment. Since this leads to an infinitely deep structure, it is not achievable for practical manufacturing.

2 https://www.technipfmc.com/

3 https://en.wikipedia.org/wiki/Comma-separated_values
